# Pathological study and molecular detection of zoonotic diseases in small ruminants at slaughterhouses in Mymensingh, Bangladesh

**DOI:** 10.14202/vetworld.2022.2119-2130

**Published:** 2022-09-05

**Authors:** Nazneen Sultana, Munmun Pervin, Sajeda Sultana, Mahmuda Islam, Moutuza Mostaree, Mohammad Abu Hadi Noor Ali Khan

**Affiliations:** Department of Pathology, Faculty of Veterinary Science, Bangladesh Agricultural University, Mymensingh, Bangladesh

**Keywords:** pathology, polymerase chain reaction, small ruminants, zoonotic diseases

## Abstract

**Background and Aim:**

Slaughterhouses act as a significant public health hotspot in developing countries like Bangladesh. The study aimed to investigate small ruminants at slaughterhouses for pathological study and molecular detection of important zoonotic diseases.

**Materials and Methods:**

A total of 75 goats and 14 sheep were investigated from June 2019 to January 2020 at different slaughterhouses in Mymensingh division, Bangladesh. The targeted diseases were tuberculosis (TB), listeriosis, Q fever, brucellosis, anthrax, toxoplasmosis, hydatidosis, and linguatulosis. The tentative diagnosis was made based on gross and histopathological lesions. Polymerase chain reaction (PCR) was performed to confirm the causal agents of zoonotic diseases using disease-specific primers.

**Results:**

Grossly, caseous nodule formation in the visceral organs; enlarged and calcifications of mesenteric lymph nodes (MLNs); hydatid cyst formation in the liver were the predominant lesions observed. Histopathologically, granuloma, caseous necrosis, and calcifications admixed with acid-fast bacteria in the MLNs, liver, spleen, and kidney were seen as suggestive of infectivity due to TB. Septic lymphadenitis mixed with rod-shaped bacteria, doughnut granuloma, fibroplasia accompanied by eosinophils and lymphocytic infiltration in MLNs, and portal granuloma were observed in listeriosis, Q fever, linguatulosis, and toxoplasmosis suspected cases, respectively. The PCR amplified *Mycobacterium tuberculosis complex* (372 bp), *Mycobacterium bovis* (600 bp), *Listeria monocytogenes* (517 bp), *Toxoplasma gondii* (512 bp), and *Coxiella burnetii* (687 bp) species-specific amplicons. In addition, linguatulosis and hydatidosis were identified in six and three goats, respectively. Brucellosis and anthrax were not detected in any cases. The slaughterhouse samples were also found to harbor the coexistence of different zoonotic pathogens.

**Conclusion:**

Deadly infectious zoonotic diseases in goats and sheep at slaughterhouses may cause widespread public health risks. As a result, more intensive monitoring and epidemiological surveys are required to successfully prevent and control zoonotic diseases.

## Introduction

Small ruminants (goats and sheep) represent Bangladesh’s economically important and potential animal resource. There are approximately 14.8 million goats and 1.9 million sheep in Bangladesh [1], which play a significant role in the economy and provide animal protein for human consumption. However, they also serve as carriers of the transmission of different infectious diseases. The most important infectious diseases such as tuberculosis (TB), listeriosis, Q fever, brucellosis, anthrax, toxoplasmosis, hydatidosis, and linguatulosis may be common in small ruminants as well as have zoonotic importance [2–5]. Zoonotic diseases are those infectious diseases that can be naturally transmitted from animals to humans. Humans are affected by approximately 1415 pathogens, among which about 61% of all pathogens are zoonotic [6]. Humans can get these zoonotic diseases from infected animals due to close contact, contaminated food or water, inhalation, and arthropod vectors (including flies, ticks, and mosquitoes).

Nowadays, livestock-associated infectious diseases are causing a significant public health impact and may lead to considerable economic losses in the agricultural sectors [7]. It also leads to significant human health hazards in developing countries like Bangladesh, where humans commonly live close to their livestock due to animal husbandry practices. Close contact is the primary mode of transmission for the majority of zoonotic diseases found in animals. As a result, people closely connected with these animals, including breeders, farmers, veterinarians, and slaughterhouse workers, are at high risk of zoonotic diseases [8]. However, in addition to close contact, urbanization and climate change can also modify the life cycle, epidemiology, and survival of vectors, which may lead to the threat of vector-borne zoonotic diseases like Q fever globally. Not only that, nowadays, human eating behavior can contribute significantly in the future to increasing zoonotic diseases [8]. On the other hand, neglected standard procedures at slaughterhouses may lead to the transmission of zoonotic diseases in developing countries like Bangladesh. As a result, obtaining safe animal food is a big problem nowadays. Therefore, preventing and controlling zoonotic pathogens that originate in livestock is important in safeguarding safe food production. There is still a lack of accurate data on the spread of zoonotic pathogens in small ruminants. In these circumstances, an accurate diagnosis of zoonotic pathogens transmitted by small ruminants is essential.

The study aimed to investigate small ruminants at slaughterhouses for pathological study and molecular detection of important zoonotic diseases.

## Materials and Methods

### Ethical approval

The study was approved by Ethical Standard of Research Committee, Bangladesh Agricultural University, Mymensingh (Approval no. BAURES/ESRC/VET/05, dated May 11, 2019).

### Study period and location

The study was conducted from June 2019 to January 2020. The samples were collected from different slaughterhouses in Mymensingh division. The samples were processed at Department of Pathology, Faculty of Veterinary Science, Bangladesh Agricultural University, Mymensingh.

### Sample collection and observation of gross pathology

A total of 75 goats and 14 sheep were investigated at various slaughterhouses in the Mymensingh division. The detailed history of slaughtered goats and sheep could not be acquired due to a lack of thorough antemortem examination in slaughterhouses in Bangladesh. However, a detailed postmortem examination was carried out to grossly diagnose diseases found in affected goats and sheep. The gross tissue changes were observed and documented. In particular, the visceral organs, including the lungs, liver, spleen, kidney, and mesenteric lymph nodes (MLNs), were examined grossly to identify any pathological changes immediately after slaughtering, both externally and internally, by visualization, palpation, and incision of the organ. After gross examination, the samples were sent to the laboratory for histopathological, parasitological, and molecular study. For the histopathological analysis, tissues from the MLNs, liver, lungs, spleen, and kidneys were collected in 10% neutral buffered formalin. In addition, parts of the tissue samples were collected aseptically in cryotubes and stored at −20°C for molecular study. Besides this, MLNs were examined for the presence of *Linguatula serrata*.

### Examination of samples for linguatulosis

To recover the nymphal stages of *L. serrata*, the lymph nodes were cut into small pieces and immersed in phosphate-buffered saline for 2 h in a Petri dish. The nymphs were collected using a dropper and examined under a microscope with a low-power objective (10×), and stored in 70% glycerin alcohol as per standard procedure [9, 10].

### Histopathological study

The formalin-fixed tissue samples were trimmed, processed, cut into 4–5 mm thick paraffin sections by microtome, and stained with routine H and E staining to observe different histopathological changes. Furthermore, Goldner’s trichrome staining was used to visualize connective tissues, notably collagen, as blue color in tissue sections. In addition, acid-fast staining was performed to observe acid-fast bacteria in tissue sections as per standard procedure [11]. The stained tissue sections were then examined at low- (10×) and high (40× and 100×)-power microscopic fields after mounting using dibutylphthalate polystyrene xylene and air drying. The images were collected on an electronic device using a microphotographic method (Cell Bioscience, Alphaimager HP, California, USA).

### DNA extraction from tissue samples

Commercially available DNA extraction kit (Wizard Genomic purification kit, Promega, USA) was used to extract microbial DNA from MLNs, liver, lungs, spleen, and kidney as per manufacturer’s instruction for the molecular detection of *Mycobacterium tuberculosis* complex (MTBC), *Mycobacterium bovis*, *Mycobacterium tuberculosis*, *Listeria monocytogenes*, *Coxiella burnetii*, *Brucella melitensis*, *Bacillus anthracis*, and *Toxoplasma gondii* by polymerase chain reaction (PCR). The purity and concentration of extracted DNA were determined using a NanoDrop™ spectrophotometer at 260 nm/280 nm (IAEA, Seibersdorf, Vienna). All of the DNA samples tested in spectrophotometry had a relatively high concentration, with a range of 250–350 ng DNA/μL volume and the extracted DNA appeared to be pure, with an A260/A280 ratio of 1.7–1.9. The extracted DNA was stored at −20°C for PCR detection of diseases.

### PCR detection of diseases

The oligonucleotide primers used in this study are given in Table-1 [12–20]. The PCR was carried out at 25 μL reaction volume using Master Mix (OneTaq^®^ Quick-Load^®^ 2X Master Mix with standard buffer, New England Biolabs, United States of America) following the manufacturer’s instructions. A thermal cycler (ProFlex gradient PCR, United States of America) was used to perform the PCR reaction. Thermal profiles of the PCR protocols designed to detect the causative agents of zoonotic diseases were carried out in 30 cycles using initial denaturation at 94°C for 30 s followed by denaturation at 94°C for 30 s; annealing at 56°C (*MPB83* gene), 58.5°C (*B1* gene), 55°C (*Inlc* gene), 59°C (*IS1111* gene), and 55°C (*IS711* gene) for 1 min, respectively, 62°C for 2 min (*16S rRNA* gene of MTBC), 57°C (*H37RvHP* gene), and 52°C (*PA* gene) for 1.5 min, respectively; extension at 68°C for 5 min; and final extension was carried out at 72°C for 5 min. The electrophoresis (WSE-1710Submerge-Mini2322100, China) was carried out on a 1.5% agarose gel containing ethidium bromide (0.5 g/mL), and images were captured using a transilluminator (Alpha imager, USA). The size of amplicons on agarose gels was determined using a 100 bp DNA ladder (TackIT, Invitrogen, USA).

**Table-1:** Oligonucleotide primers used in PCR detection of zoonotic diseases in slaughtered small ruminants.

Genes targeted	Primers	Sequences (5′-3′)	Organism	Amplicon size	References
*IS711*	BMF	aaatcgcgtccttgctggtctga	*Brucella melitensis*	731 bp	[12, 13]
	BMR	tgccgatcacttaagggccttcat			
*B1*	ToxoplasF1	ctctctgttacacgctctcag	*Toxoplasma gondii*	512 bp	[14]
	ToxoplasR1	cgaacacagcgtgttcttgcc			
*Inl C*	LMF	aattcccacaggacacaacc	*Listeria monocytogenes*	517 bp	[15]
	LMR	cgggaatgcaatttttcacta			
*16S rRNA*	TB 1-F	gaacaatccggagttgacaa	*Mycobacterium tuberculosis complex*	372 bp	[16]
	TB 1-R	agcacgctgtcaatcatgta			
*MPB83*	MPB83F	cagggatccaccatgttcttagcgggttg	*Mycobacterium bovis*	600 bp	[17]
	MPB83F	tggcgaattcttactgtgccggggg			
*H37RvHP*	H37RvHPF	gaactcaccgtcggtggtga	*Mycobacterium tuberculosis*	667 bp	[18]
	H37RvHPR	ccttgctcgatctctgcgtc			
*PA*	PA8	gaggtagaaggatatacggt	*Bacillus anthracis*	596 bp	[19]
	PA5	tcctaacactaacgaagtcg			
*IS1111*	CoxBurF1	tatgtatccaccgtagccagt	*Coxiella burnetii*	687 bp	[20]
	CoxBurR1	cccaacaacacctccttattc			

PCR=Polymerase chain reaction

## Results

In this study, out of 75 goats and 14 sheep investigated, TB, listeriosis, Q fever, linguatulosis, hydatidosis, and toxoplasmosis were tentatively diagnosed by gross and histopathological examination. Further, the molecular detection technique (PCR) confirmed TB in 19 goats and one sheep, listeriosis in three goats and four sheep, Q fever in one goat, and toxoplasmosis in two goats. Among them, TB was detected relatively higher in number than other organisms in goats, and listeriosis was detected in higher numbers than other organisms in sheep. However, brucellosis and anthrax were not identified in goats, and brucellosis, anthrax, linguatulosis, hydatidosis, toxoplasmosis, and Q fever were not detected in sheep. Overall, the number of detected diseases was relatively higher in goats than in sheep. In addition, coinfection of TB with toxoplasmosis, TB with Q fever, and TB with listeriosis were detected in two, one, and one goat, respectively. The overall percentages of zoonotic diseases in goats and sheep are given in Table-2.

**Table-2:** Percentages of zoonotic diseases in goats (n = 75) and sheep (n = 14).

Targeted diseases	Percentages of infection	

	Goat	Sheep
Tuberculosis	(25.33%, 19/75)	(7.14%, 01/14)
Listeriosis	(4%, 03/75)	(28.57%, 04/14)
Q fever	(1.33%, 01/75)	0.00
Linguatulosis	(8%, 06/75)	0.00
Hydatidosis	(4%, 03/75)	0.00
Toxoplasmosis	(2.67%, 02/75)	0.00
Brucellosis	0.00	0.00
Anthrax	0.00	0/00

### Tuberculosis

**Figure-1:**
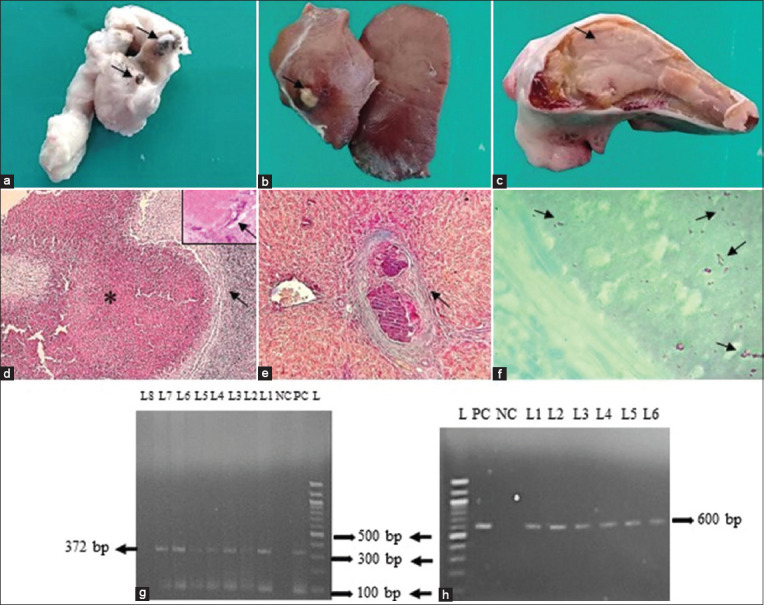
Examination of the mesenteric lymph node (MLN), liver, and spleen of tuberculosis-infected small ruminants. (a) Calcifications (arrows) were seen at the cut surface of the MLN. (b) A caseous nodule (arrow) was seen on liver. (c) Semisolid caseous mass (arrow) was seen at the cut surface of the spleen. (d) Caseous necrosis (asterisk) surrounded by fibrous connective tissues (arrow) and infiltrated with inflammatory cells predominantly lymphocytes, macrophages and Langhan’s types giant cells (inset: Higher magnification) were seen in the MLN. (e) Calcification encapsulated by blue color fibrous connective tissue proliferation (arrow) was seen in the liver. (f) Pink color rod-shaped acid-fast bacilli (arrows) were seen in the caseous center of MLN. H & E staining (d), Goldner’s trichrome staining (e), and acid-fast staining (f). (d-e = 10×; inset (d) and f = 100×). (g) PCR amplified products of 372 bp fragments of the *16S rRNA* gene of *Mycobacterium tuberculosis* complex isolates from small ruminants. L = DNA marker (100 bp), PC = Positive control, NC = Negative control, lane 01–07 = representative *M. tuberculosis* complex isolates from the MLNs and liver of small ruminants. (h) PCR amplified products of 600 bp fragments of the *MPB83* gene of *Mycobacterium bovis* isolates from small ruminants. L = DNA marker (100 bp), PC = Positive control, NC = Negative control, lanes 01–06 = representative *M. bovis* isolates from the liver and MLNs of small ruminants.

Grossly, TB-infected animals showed caseocalcified lesions in lymph nodes and visceral organs. Lesions in the MLNs were characterized by semisolid cheesy caseous mass and calcified gritty material development after the cross-section of the MLNs (Figure-1a). In the liver, granuloma encapsulated with fibrous connective tissues was seen (Figure-1b). Tubercle-like lesions were observed in the lungs. Lesions in the spleen revealed semisolid yellowish caseous materials formation at the cut surface (Figure-1c). In addition, a caseous nodule was also seen in the kidney of one goat. Histopathological investigation of TB-infected animals showed granuloma formation in visceral organs. The granuloma comprised macrophages, a few lymphocytes, and epithelioid cells predominantly. In addition, caseous necrosis encapsulated with fibrous connective tissues accompanied by infiltration of macrophages and Langhan’s types of giant cells was seen in the liver, spleen, kidney, and MLNs (Figure-1d). Calcifications encapsulated by fibrous connective tissue proliferation were also observed in the MLNs, liver, and spleen. In this study, blue-colored fibrous connective tissue proliferation was distinctly visualized by Goldner’s trichrome staining (Figure-1e). Acid-fast staining of the tissue sections showed the presence of acid-fast bacteria in the epithelioid cells of the macrophages and caseous necrosed area (Figure-1f). To confirm TB, the extracted DNA (MLNs, liver, lungs, kidney, and spleen) was used to amplify fragments of the *16S rRNA* gene of MTBC (372 bp) (Figure-1g) and was found positive in 19 goats and one sheep.

Further, PCR was performed to amplify fragments of the *MPB83* and the *H37RvHP* genes to detect the infectivity due to *M. bovis* (600 bp) and *M. tuberculosis* (667 bp), respectively. Finally, in 10 goats and one sheep, *MPB83* gene-specific amplification of genomic DNA was generated in the MLNs, liver, lungs, spleen, and kidney (Figure-1h). However, *M. tuberculosis* (*H37RvHP* gene) was not detected in any cases.

### Listeriosis

**Figure-2:**
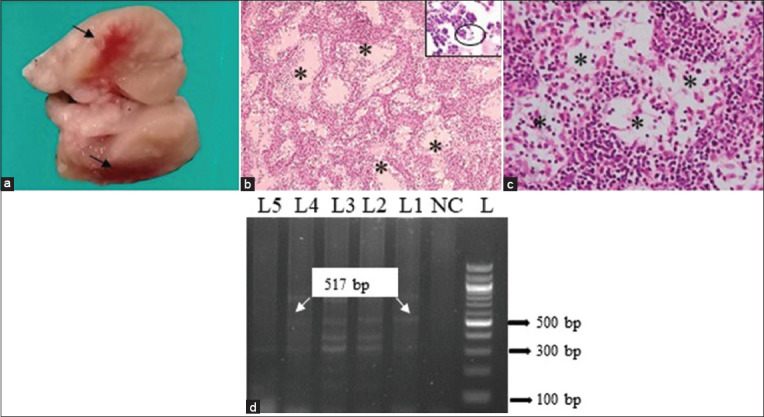
Examination of mesenteric lymph nodes (MLNs) of *Listeria monocytogenes* infected small ruminants. (a) Hemorrhages (arrows) were seen at the cut surface of MLN of goat. (b) Septic lymphadenitis (asterisks) and the presence of rod-shaped bacteria (circle) with infiltration of band neutrophils (inset in higher magnification) were seen in the MLN of goats. (c) Marked lymphoid depletion (asterisks) was seen in MLN of sheep. H & E staining (b and c). (b = 10×, inset (b) and c = 40×). (d) PCR amplified products of 517 bp fragments of the *Inlc* gene of *L. monocytogenes* isolates from goats. L = DNA marker (100 bp), NC= Negative control, lanes 1–4 = representative *L. monocytogenes* isolates from the MLN of small ruminants.

In *Listeria* suspected cases, MLNs were grossly enlarged, edematous, and hemorrhagic (Figure-2a). Histopathologically, septic lymphadenitis was admixed with rod-shaped bacteria accompanied by infiltrates of inflammatory cells, mainly neutrophils and lymphocytes (Figure-2b). In addition, marked lymphoid depletion was also seen in the affected lymph nodes (Figure-2c). To confirm listeriosis, PCR was performed to detect the *InlC* gene of *L. monocytogenes* (517 bp) and an *InlC* gene-specific amplicon was generated in the MLNs of three goats and four sheep (Figure-2d).

### Q fever

**Figure-3:**
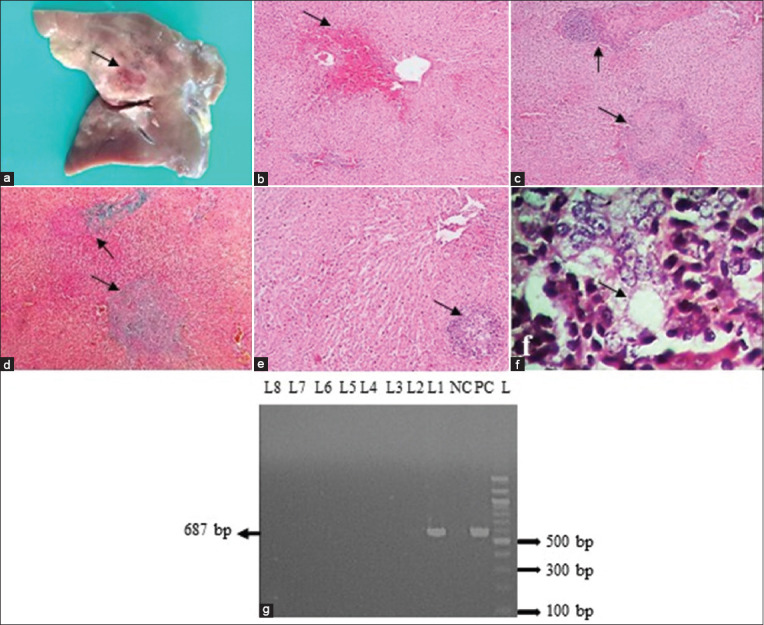
Examination of liver of *Coxiella burnetii-*infected goat. (a) Hemorrhages (arrow) were seen on liver. (b) Extensive hemorrhages (arrow) were seen around the central vein of liver. (c-d) Extensive fibrin mimicking the necrotizing granuloma (arrows) was seen. (e and f) Typical fibrin ring (doughnut shape) granuloma (arrow) was seen, which was characterized by a centrally lipid vacuole surrounded by a fibrin ring accompanied by infiltrates with macrophages and lymphocytes. H & E staining (b and c, e and f); Goldner’s trichrome stain (d). (b–e = 10×, f = 100×). (g) PCR amplified products of 687 bp fragments of the *IS1111* gene of *C. burnetii* isolates from goats. L = DNA marker (100 bp), PC= Positive control, NC= Negative control, lane 1 = *C. burnetii* isolates from the liver of goat.

Grossly, hepatomegaly and splenomegaly were seen in Q fever infection. In addition, hemorrhages were seen on the parietal surface of the liver (Figure-3a). Histopathologically, lesions consistent with Q fever revealed widespread hemorrhages in the liver (Figure-3b). Widespread fibrin that resembled necrotizing granuloma but lacked typical fibrin ring formation was seen (Figure-3c and d). Typical fibrin ring (doughnut-shaped) granuloma characteristics of Q fever were also seen (Figure-3e and f). The granuloma was mainly comprised of lymphocytes and macrophages (Figure-3f). In addition, the lesions in the spleen revealed hemorrhagic splenitis, which was accompanied by infiltrates of neutrophils and macrophages, as well as the presence of bacterial colonies in and around the macrophages. PCR was used to amplify the fragments of the *IS111* gene of *C. burnetii* and was found positive in the liver of one goat (Figure-3g). However, the *IS111* gene-specific amplicon was not generated in sheep.

### Linguatulosis

**Figure-4:**
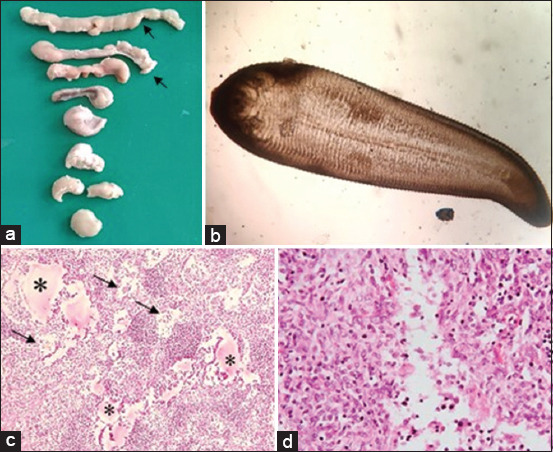
Examination of mesenteric lymph nodes (MLNs) of *Linguatula serrata-*infected goats. (a) Enlarged MLNs (arrows) were seen. (b) Observation of *Linguatula serrata* under a microscope. (c) Edema (asterisks) and lymphoid depletion (arrows) were seen. (d) The proliferation of fibroblast cells and infiltration of eosinophils were seen. H & E staining (c and d); (b and c = 10×, d = 40×).

Grossly, enlarged, soft, and hemorrhagic MLNs were seen in linguatulosis (Figure-4a). The nymphal stage of *Linguatula* was visualized under the microscope in six goats (Figure-4b). However, *Linguatula* was not identified in sheep. Histopathologically, edema, lymphoid depletion, and proliferation of fibroblast cells with inflammatory cells were seen (Figure-4c and d).

### Hydatidosis

**Figure-5:**
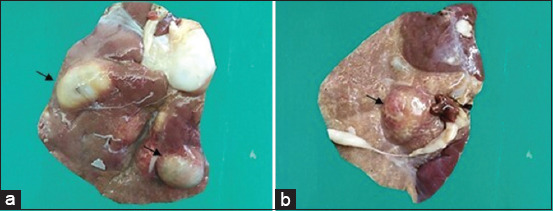
Examination of liver of hydatidosis-infected goats. (a and b) Hydatid cysts were seen on the parietal surface of the liver (arrows).

Grossly, single to multiple cystic developments were seen in three goats’ liver (Figure-5). Histopathologically, widespread cirrhosis was observed.

### Toxoplasmosis

**Figure-6:**
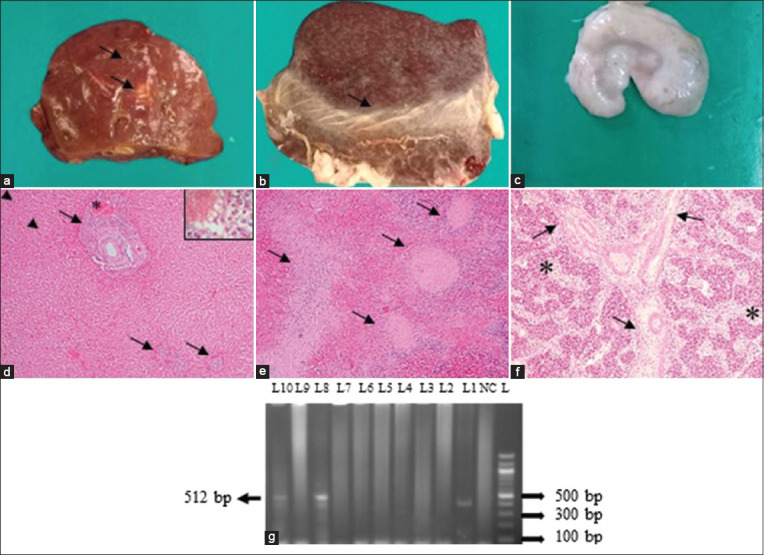
Examination of liver, spleen, and mesenteric lymph node (MLN) of *Toxoplasma gondii-*infected goat. (a) Necrotic lesions (arrows) were seen on liver. (b) Fibrinous covering (arrow) was seen on spleen (c) Swollen MLN was seen. (d) Multifocal portal granuloma (arrows) was seen which was characterized by infiltration of lymphocytes and macrophages (inset in higher magnification), congestion in a central vein (asterisk), as well as sinusoidal hemorrhages and pyknosis of hepatocytes (arrow heads), were seen. (e) Hyperplasia of trabeculae (arrows) of spleen was seen. (f) Lymphoid depletion (asterisks) with enlargement and branching of trabeculae (arrows) were seen. H & E staining (d–f). (d–f = 10×, inset [d] = 100×). (g) PCR amplified products of 512 bp fragments of the *B1* gene of *T. gondii* isolates from goats. L = DNA marker (100 bp), NC = Negative control, lane (8, 10) = representative *T. gondii* isolates from the liver and spleen of goat.

Grossly, cirrhotic liver, swollen spleen, and MLNs were suggested to be *Toxoplasma* infections (Figure-6a–c). Histopathologically, lesions consistent with *Toxoplasma* infection revealed multifocal portal granuloma accompanied by macrophages and lymphocytes infiltration, congestion in the central vein, pyknosis of hepatocytes, and hemorrhages in sinusoids (Figure-6d). Lymphoid depletion with trabecular enlargement of MLNs and in the spleen and hyperplasia of the trabeculae was observed (Figure-6e and f). PCR was performed to detect the fragments of the *B1* gene of *T. gondii*. Finally, *B1* gene-specific amplicon was generated in two goats (Figure-6g). However, it was not detected in sheep.

### Brucellosis

Grossly, lymphadenopathy was suspected as brucellosis. To confirm brucellosis, PCR was performed targeting the *IS1111* gene of *B. melitensis* (731 bp). This gene, however, was not generated in any cases in this study.

### Anthrax

Grossly, splenomegaly was considered an anthrax suspect case. To confirm, PCR was performed targeting the *PA* gene of *B. anthracis* and was not generated in any suspected cases.

## Discussion

The study aimed to investigate small ruminants at slaughterhouses for pathological study and molecular detection of important zoonotic diseases. Small ruminants (goats and sheep) can serve as reservoirs for pathogens that can cause domestic zoonoses and play a crucial role in transmitting diseases to people. Zoonotic diseases not only make animals sick but also represent a serious threat to human health. Anthrax, TB, listeriosis, brucellosis, Q fever, toxoplasmosis, linguatulosis, hydatidosis, etc., are zoonotic diseases that can be transferred to humans from small ruminants.

In this study, we identified three different bacterial diseases (TB, listeriosis, and Q fever) and one protozoal disease (toxoplasmosis) by gross and histopathological lesions and molecular detection technique (PCR). In addition, two parasitic diseases (linguatulosis and hydatidosis) were identified by pathological examination. Among them, TB was detected relatively higher in number (19/75) than other organisms in goats, and listeriosis was detected in higher number (04/14) than other organisms in sheep. However, brucellosis and anthrax were not identified in goats and brucellosis, anthrax, linguatulosis, hydatidosis, toxoplasmosis, and Q fever were not detected in sheep. Overall, the number of detected diseases was relatively higher in goats than in sheep, possibly due to smaller sample sizes or the fact that sheep can act as natural resistance to many infectious diseases [21].

Tuberculosis is a chronic disease that causes exudative granulomatous inflammatory lesions in the lungs, lymph nodes, and visceral organs. The disease affects a wide range of hosts and significantly impacts veterinary and human health. Globally, TB in livestock causes significant economic losses due to decreased production and early culling of potentially high-yielding animals and can affect the productivity of small ruminants in many parts of the world [22, 23]. Tuberculosis is caused by acid-fast bacteria of the members of the MTBC. However, in small ruminants, TB is primarily caused by two MTBC members (*M. bovis* or *Mycobacterium caprae*) [24], while infection with *M. tuberculosis* and *Mycobacterium* *avium* has also been observed [25]. *M. bovis* is the most common *Mycobacterium* pathogen, affecting many animals, including humans and small ruminants, which can act as reservoirs for *M. bovis*.

In this study, encapsulated caseocalcified lesions were seen in the MLNs and spleen. The lungs, liver, and kidney also noted caseous inflammatory lesions. Tuberculosis lesions in small ruminants are mainly similar to cattle lesions [26]. In goats infected with *M. bovis*, postmortem examination commonly reveals circumscribed pale yellow, white, caseous, or caseocalcareus lesions of various sizes, often encapsulated, particularly in the lungs and mediastinal lymph nodes or in the MLNs, according to Sharpe *et al*.[24]. However, this study observed no differences in sheep and goat lesions. In this study, caseocalcified lesions mainly were found in MLNs, followed by the liver, spleen, lungs, and kidney, all of which are suggested to have been infected by both ingestion and inhalation [22, 27].

Histopathologically, the lesions revealed central caseous necrosis with or without calcifications, infiltrated with macrophages, epithelioid cells, lymphocytes, and Langhan’s types of giant cells surrounded by fibrous connective tissues in the MLNs, liver, and spleen. Granulomatous inflammation was also observed in the liver, lungs, and kidneys. Mostly, the granuloma was comprised of macrophages, lymphocytes, and Langhan’s types of giant cells. The findings agreed with the research of Aljameel *et al*. [27]. Few acid-fast bacilli were seen in the caseous necrosed area, epithelioid cells of macrophages, and in Langhan’s giant cells [28].

The polymerase chain reaction was used to confirm the specific causal agents of the TB and successfully detected the *16S rRNA* gene of MTBC in 19 goats and one sheep. Further, PCR was performed to amplify fragments of the *MPB83* and the *H37RvHP* genes to detect the infectivity due to *M. bovis* (600 bp) and *M. tuberculosis* (667 bp), respectively. Finally, amplification of MPB83 gene was generated in ten goats and one sheep. However, *M. tuberculosis (H37RvHP* gene) was not detected in any cases. Tuberculosis in small ruminants is predominantly caused by two members of the MTBC *(M. bovis* or *M. caprae*) [29]. Here, we also detected the *MPB83* gene of *M. bovis*, but *M. caprae* strain could not be identified due to the limitation of the diagnostic technique. Hence, further research should be done to identify *M. caprae* strain. In Bangladesh, there is a scarcity of information on the molecular detection of TB in small ruminants. In Bangladesh, bovine and avian TB have previously been detected in small ruminants using the comparative cervical tuberculin and caudal fold test [30]. However, these techniques are rarely used in small ruminant farming due to the thinness of the skin. Furthermore, lesion detection and bacterial culture in live animals do not provide significant results [31]. As a result, the discovery of technology for detecting TB in live small ruminants is a big problem, and detecting lesions or tuberculous organisms during postmortem examination, followed by final confirmation by PCR, is preferred.

Small ruminants were assumed initially to play a modest part in TB epidemiology due to their resistance to the disease or their position as a spillover host [21]. Numerous studies have recently suggested that sheep and goats can be infected with TB due to close contact with diverse species, a low biosecurity status of the farming system, and a high TB infection rate [23, 29]. As bovine TB is endemic in Bangladesh [18], and mixed farming is widespread in Bangladesh, there is a risk of TB transmission to goats and sheep, as shown in this study. These findings suggest that *M. bovis* infection in goats and sheep is rising in Bangladesh, and goats may serve as a TB reservoir. *Mycobacterium bovis* is a zoonotic pathogen that can silently spread bovine TB to other vulnerable animals during slaughter. In the grazing field, it is indispensable to take actions to prevent and eradcate TB.

Listeriosis is one of the most serious food-borne bacterial diseases caused by *L. monocytogenes*, a facultative anaerobe, a Gram-positive bacillus that can thrive in various temperatures, including cold (4–10°C). *L. monocytogenes* is a saprophyte found in soil, standing water, animal feeds (silage), and the meat products produced by these animals. Although it is extensively distributed in the environment, it can cause serious invasive sickness in livestock and humans [32]. In Bangladesh, *L. monocytogenes* was previously isolated from 16.66% (2/16) beef and 8.33% (1/12) chevon by bacteriological identification methods in Mymensingh municipality [3]. *L. monocytogenes* has also been identified in different parts of the world. In Egypt, virulence genes (*hlyA*, *iap*, and *actA*) of *L. monocytogenes* were detected in various food products [33]. The prevalence of listeriosis in chevon (9.8%) and beef (8.9%) has been reported in North East India [34].

Grossly, in this study, the lesions suggestive of *Listeria* infected goats and sheep showed marked lymphadenomegaly and hemorrhages in MLNs. Similar findings were reported earlier in the case of listeriosis-infected animals [35]. Histopathologically, infected MLNs showed septic lymphadenitis with exudates containing neutrophils and mononuclear cells [36]. In addition, depletion of lymphocytes with widened trabeculae was seen, which supported the findings of Fairley *et al*. [37]. This study restricted lesions to the MLNs without involving the other visceral organs. In the case of food-borne listeriosis, after colonization of bacteria in the intestinal villi, the bacteria multiply continuously and shed into the small intestinal lumen, where they locate in the nearby MLNs where the bacteria then disseminate to the visceral organs through the MLNs [38]. As a result, MLNs can serve as a vehicle for spreading the infection to the visceral organs. In this study, to confirm *L. monocytogenes*, *InlC* gene (517 bp) of *L. monocytogenes* has been amplified by PCR. Finally, the PCR amplified 517 bp amplicons in three goats and four sheep. Previously, the *InlC* gene has been successfully amplified to detect *L. monocytogenes* from different food samples [33]. Law *et al*. [39], reported that molecular detection is a quick test for detecting *L. monocytogenes* in a variety of foods, including animal-derived foods.

Another significant bacterial zoonotic disease is Q fever, caused by *Coxiella burnetii*, Gram-negative obligate intracellular bacteria belonging to the Legionellales order. Q fever is an OIE-listed notifiable disease and a ubiquitous zoonotic disease reported worldwide except in New Zealand. Cattle, sheep, and goats are the primary reservoirs of Q fever. However, other mammals, including humans, have been infected with the disease.

Grossly, in this study, the lesions suggestive of Q fever-infected goats exhibited hepatomegaly and splenomegaly [40]. Histopathologically, a characteristic fibrin ring granuloma (doughnut granuloma) with central lipid cores surrounded by a fibrin ring and infiltration of inflammatory cells, mainly lymphocytes and macrophages, was seen, and this lesion was the most striking finding of this study. Previously, similar lesions were observed in patients infected with Q fever [41, 42]. However, information is lacking about Q fever hepatitis in small ruminants. While fibrin ring granuloma is not pathognomonic for Q fever diagnosis since the lesion may be seen in other diseases [41], it may serve as a warning indication of Q fever. In addition, widespread fibrin resembling necrotizing granuloma but lacking typical fibrin ring formation was seen [41, 42]. In the spleen, the lesions revealed hemorrhagic splenitis, which was accompanied by infiltrates of neutrophils and macrophages, as well as a bacterial colony in and around the macrophages, suggesting that macrophages play a major role during the C. burnetii infection.

A polymerase chain reaction was carried out to confirm Q fever using one set of primers for amplifying fragments of the *IS1111* transposon-like repetitive element of *C. burnetii* and found positive in one goat. The *IS1111* gene was used previously by other researchers to detect *C. burnetii* [43]. Das *et al*. [44] reported that *IS1111* is the best-known target to detect *C. burnetii* in an active infection. Several serological tests are also available at the herd level to detect *C. burnetii* antibodies [45]. Previously, Rahman *et al*. [45] reported the seroprevalence of Q fever in sheep (9.52%), goats (3.33%), and cattle (3.57%) in different districts of Bangladesh. They also reported that 4.35% of sheep fetal membranes were positive in rt PCR, however, no positive cases were found in sheep in this study. This may be due to sampling limitations. A higher prevalence of Q fever was reported in different countries. In Egypt, the molecular prevalence of Q fever in sheep and goats’ raw milk was 82.4% and 89.5%, respectively [46]. The seroprevalence of Q fever was identified in 25.68% and 28.20% of sheep and goats, respectively, in El Minya Governorate, Egypt, between August 2016 and January 2017 [47]. Previously, Q fever was reported in 25.71–57.1% of farm workers in Egypt [46, 47], so the public health significance of Q fever should be considered.

Linguatulosis, caused by *L. serrata*, is a food-borne helminth-like endoparasite that affects a wide range of animals, including humans. Herbivores and humans that act as intermediate hosts are particularly vulnerable to this infection. *L. serrata* infection has been reported worldwide, including India [48] and Bangladesh [9]. Previously, linguatulosis in the Mymensingh district of Bangladesh has been reported in 31% of goats and 50.7% of cattle [4]. Human infection with *L. serrata* has already been found in Malaysia [49] and India [50]. As a result, the importance of linguatulosis in terms of public health should not be neglected.

Grossly, in this study, the *Linguatula-*infected goats showed enlarged, soft, and hemorrhagic lymph nodes, as described earlier by Hajipour and Tavassoli [51]. The presence of honeycomb appearances of the MLNs was previously reported in the case of *L. serrata* infection [4], but was not observed in this study. Infected MLNs exhibited inflammatory lesions, edema, lymphoid depletion, fibroplasia, and mononuclear cell infiltration. Similar lesions in the MLNs of goats infected with *L. serrata* were reported at the chronic stage of linguatulosis [52]. Severe damage to the parenchyma of the MLNs with massive proliferation of fibrous tissues was seen associated with eosinophilic infiltration [4]. Eosinophils mainly release various toxins such as eosinophil cationic proteins to kill the parasites during parasitic infection.

Hydatidosis, caused by *Echinococcus granulosus*, is a serious zoonotic parasitic disease affecting a wide range of animals where domestic and wild carnivores act as definitive hosts, and domestic hosts such as livestock are the major reservoirs for the final host. The diseases can affect different organs of the body, but the liver and lungs are most commonly affected [53]. Hydatidosis contributes mainly to liver condemnation and economic losses [54].

Grossly, in this study, single to multiple cysts were seen in three goat livers that ranged in size, protruding from the surface of the liver, soft to the touch, and containing faint turbid fluids [2]. Previously, caprine hydatidosis was reported (4.94%) in the Chittagong district of Bangladesh [2]. Different levels of hydatidosis have been reported in various countries around the world. In Iran, the prevalence was 3.72% in cattle, 2.85% in goats, and 2.40% in sheep [55].

Toxoplasmosis is caused by *Toxoplasma gondii*, a common protozoan parasite that may infect all warm-blooded animals in developed countries; however, felids, such as domestic cats, are the only known definitive hosts. Previously, toxoplasmosis was reported at 15.52% in goats’ aborted fetuses in Bangladesh [56]. In North India, a similar prevalence was found in goats’ cardiac and skeletal muscles [57].

Grossly, in this study, *T. gondii*-infected goats showed liver cirrhosis. During *T. gondii* infection, the parasite moves to the surface of sinusoidal epithelial cells of Kupffer cells after infiltrating the liver, then eventually attaches and develops in the cytoplasm of both hepatocytes and stellate cells, which increases the number of activated hepatic stellate cells, resulting in chronic liver failure and cirrhosis [58]. In addition, MLNs were found enlarged and edematous. Histopathologically, necrotizing lymphadenitis, lymphoid depletion with mononuclear cells and macrophages infiltration, and trabecular enlargement were seen in MLNs and spleen [58]. In the liver, multifocal portal granuloma was observed in one case, with infiltration of mononuclear cells and macrophages.

In addition, mild congestion was observed in central veins and sinusoids, which may be due to the presence of *Toxoplasma* infection [59]. To confirm *T. gondii*, PCR was used to amplify fragments of the *B1* gene (512 bp) of *T. gondii* and was found positive in two goats. The *B1* gene was selected because of its frequent use in detecting *T. gondii* as reported by other researchers [56, 60].

## Conclusion

We detected three bacterial and three parasitic diseases at slaughterhouses in Mymensingh division, including TB, listeriosis, Q fever, linguatulosis, hydatidosis, and toxoplasmosis. All of which are associated with deadly zoonotic diseases in humans and animals. Among the detected diseases, TB was detected in a higher number (19/75) in goats, followed by linguatulosis (06/75) than in other organisms, and listeriosis was detected in a higher number (04/14) in sheep. The zoonotic diseases detected in slaughterhouses in this study are of great concern for entering the food chain and for their pathogenicity to humans. Pathological study and the PCR protocols adapted in this study will help detect these diseases at slaughterhouse origin. Finally, more extensive investigation is necessary to molecularly characterize these zoonotic pathogens to better understand the epidemiology of these diseases and monitor new pathogens and factors influencing their occurrence, which would help implement integrated management measures to control these pathogens in Bangladesh.

## Authors’ Contributions

NS, MP, and MAHNAK: Designed the study and prepared the manuscript for submission. NS, SS, MI, and MM: Collected and processed the samples. NS: PCR, gel electrophoresis, and histopathology. NS, MP, and MAHNAK: Drafted and revised the manuscript. All authors have read and approved the final manuscript.
